# *Bartonella henselae* mediastinal lymphadenitis mimicking malignancy with critical airway compression in a child: a case report

**DOI:** 10.3389/fped.2026.1871232

**Published:** 2026-06-24

**Authors:** Tong Lu, Sijuan Sun, Teng Teng, Jian Zhang, Qing Cao, Hong Ren

**Affiliations:** 1 Department of Pediatric Intensive Care Unit, Shanghai Children's Medical Center, School of Medicine, Shanghai Jiao Tong University, Shanghai, China; 2 Department of Infectious Diseases, Shanghai Children’s Medical Center, School of Medicine, Shanghai Jiao Tong University, Shanghai, China

**Keywords:** airway compression, *Bartonella henselae*, case report, cat-scratch disease, mediastinal lymphadenitis, metagenomic next-generation sequencing

## Abstract

Cat-scratch disease, caused by *Bartonella henselae*, is usually a self-limited infection presenting with regional lymphadenopathy in children. Thoracic involvement is uncommon, and mediastinal lymphadenitis with clinically significant airway compression may closely mimic malignancy. We report a previously healthy 6-year-old boy who presented with persistent fever, mild cough, weight loss, and cervical lymphadenopathy. Chest computed tomography revealed necrotic mediastinal lymphadenopathy forming a mass-like lesion with compression of the right middle lobe bronchus and associated atelectasis. Bronchoscopy showed severe bronchomalacia with approximately 90% luminal narrowing, despite only mild respiratory symptoms. Initial antimicrobial therapy failed to improve the clinical or radiologic abnormalities. Because of constitutional symptoms and a necrotic mediastinal mass, lymphoma was strongly suspected; however, bone marrow examination was unrevealing. During biopsy of the mediastinal lesion, purulent material was encountered. Histopathology demonstrated necrotizing granulomatous inflammation, and metagenomic next-generation sequencing identified *Bartonella henselae*, establishing the diagnosis of cat-scratch disease. Treatment with doxycycline and rifampin led to prompt resolution of fever and marked radiologic improvement, with substantial relief of airway compression. This case highlights that *Bartonella henselae* infection can present as a necrotic mediastinal mass with severe but reversible airway compression in children. Cat-scratch disease should be considered in the differential diagnosis of pediatric mediastinal masses, particularly when inflammatory features, cat exposure, and discordant respiratory symptoms are present. Integration of imaging, bronchoscopy, pathology, and molecular testing may prevent misdiagnosis as malignancy and underestimation of airway risk.

## Introduction

Cat-scratch disease is a self-limited infection caused by *Bartonella henselae* (1). In children, it most commonly manifests as a benign and self-limited illness with regional lymphadenopathy after exposure to cats. However, atypical presentations involving visceral organs, the eye, central nervous system, bone, or thorax may occur and can create substantial diagnostic uncertainty (2).

Thoracic involvement in cat-scratch disease is uncommon (3). When mediastinal lymph nodes are involved, imaging may show necrotic lymphadenopathy or mass-like lesions that mimic lymphoma, tuberculosis, fungal infection, or other granulomatous diseases (4–7). This diagnostic overlap is clinically important because children with mediastinal masses may be evaluated urgently for malignancy, and airway compression may carry serious risk.

Severe airway compression caused by *Bartonella henselae* mediastinal lymphadenitis appears to be distinctly rare in children. Moreover, the degree of airway compromise may not be fully reflected by respiratory symptoms (8). We report a child with cat-scratch disease presenting as necrotic mediastinal lymphadenitis mimicking malignancy and causing critical but reversible airway compression. This case emphasizes the value of combining clinical exposure history, cross-sectional imaging, bronchoscopy, histopathology, and metagenomic next-generation sequencing for diagnosis.

## Case description

A previously healthy 6-year-old boy presented with a 2-week history of persistent fever, mild cough, and weight loss. At admission, the patient's temperature was 36 °C, heart rate was 98 beats/min, respiratory rate was 22 breaths/min, blood pressure was 104/65 mmHg, and oxygen saturation was 97% in room air. Physical examination revealed small cervical lymph nodes and decreased breath sounds over the right lung with no obvious signs of respiratory distress. No rash or inoculation papule was observed. He appeared well nourished, although his body weight had decreased by approximately 2 kg over the preceding two weeks. Immunizations were up to date. There was no known tuberculosis contact or family history of tuberculosis.

**Figure 1:**
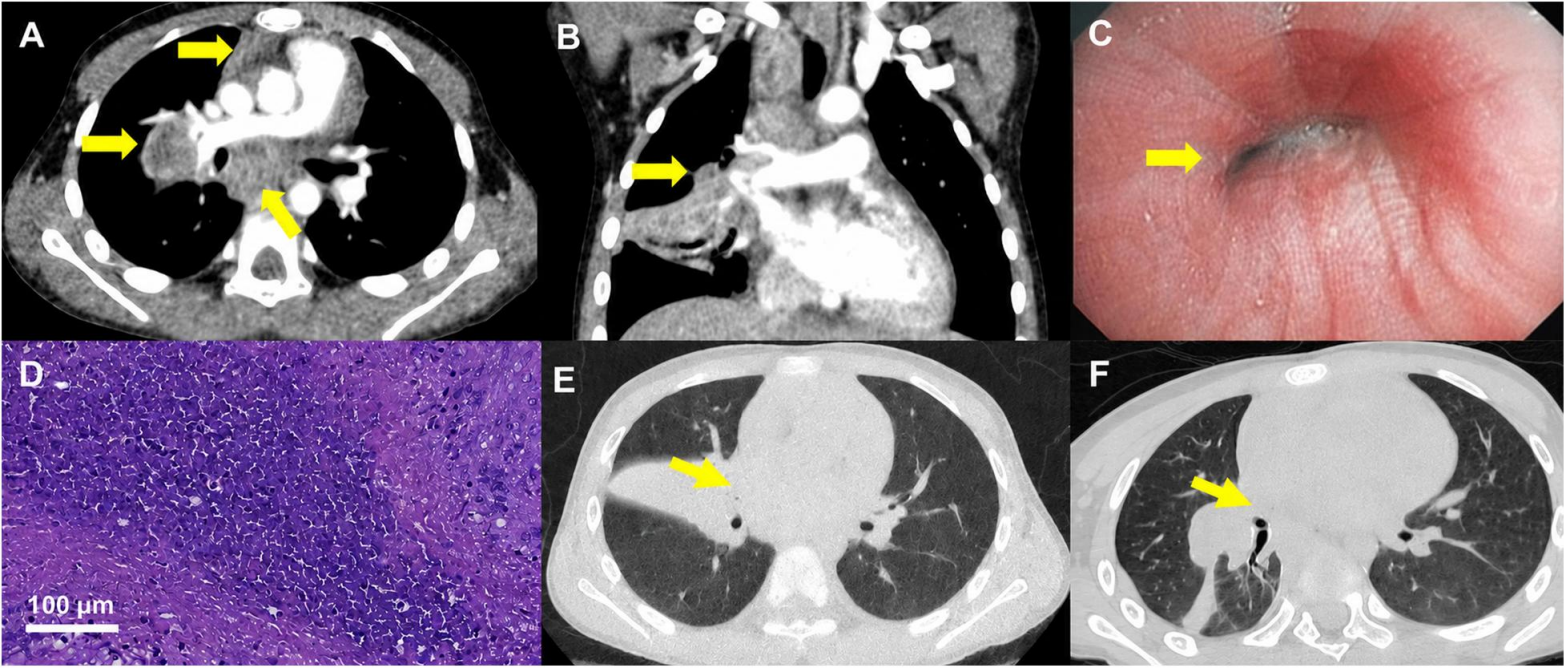
Radiologic, bronchoscopic, and histopathologic findings of *Bartonella henselae* mediastinal lymphadenitis with airway compression. **(A)** Axial chest computed tomography showing a necrotic mediastinal/hilar mass-like lesion compressing the adjacent airway (yellow arrows). **(B)** Coronal chest computed tomography showing the right-sided mediastinal/hilar lesion (yellow arrow). **(C)** Bronchoscopy demonstrating severe narrowing of the right middle lobe bronchus with marked bronchomalacia (yellow arrow). **(D)** Histopathologic examination showing granulomatous inflammation with necrosis, partial caseous necrosis, and lymphoid follicle formation. (Hematoxylin and eosin staining; scale bar, 100 μm). **(E)** Pretreatment chest computed tomography showing severe airway compression and associated pulmonary infiltrative changes (yellow arrow). **(F)** Follow-up chest computed tomography after antimicrobial treatment showing marked improvement in bronchial compression and pulmonary infiltrates (yellow arrows).

Chest computed tomography (CT) showed mediastinal lymphadenopathy forming a mass-like lesion with central necrosis. The lesion caused significant compression of the right middle lobe bronchus and was associated with atelectasis (Figures 1A,B). Bronchoscopy demonstrated severe bronchomalacia with approximately 90% luminal narrowing of the right middle lobe bronchus, suggesting impending airway obstruction (Figure 1C). Notably, the severity of airway narrowing was disproportionate to the relatively mild respiratory symptoms. Abdominal imaging, including ultrasound and CT, showed no obvious abnormalities, with no evidence of hepatosplenic involvement or intra-abdominal abscesses.

Laboratory testing showed elevated inflammatory markers, including C-reactive protein (36.7 mg/L; reference, <10 mg/L), erythrocyte sedimentation rate (59 mm/h; reference, 0–20 mm/h). The white blood cell count was 11.83 × 10^9/L (reference, 4.3–11.3 × 10^9/L), and procalcitonin was 0.06 ng/mL (reference, <0.05 ng/mL). Ferritin, lactate dehydrogenase, liver function, and renal function were within normal limits. Metagenomic testing of bronchoalveolar lavage fluid suggested *Streptococcus* species, consistent with the pre-admission microbiologic result. Empirical antimicrobial therapy with amoxicillin plus linezolid was administered for 5 days; however, the patient showed no meaningful clinical response, primarily manifested by persistent fever and persistently elevated C-reactive protein. Repeat contrast-enhanced chest CT showed no significant radiologic regression compared with the CT obtained before admission and continued to raise concern for lymphoma.

Further history revealed exposure to a household kitten and a possible scratch, although no typical inoculation lesion was identified. Because of the constitutional symptoms, necrotic mediastinal mass, and persistent imaging abnormalities, malignancy, especially lymphoma, was strongly suspected. Surgical consultation was obtained immediately. Bone marrow examination was performed first and showed no evidence of malignant infiltration. Although PET-CT was considered useful for disease assessment, it was deferred because of concerns regarding radiation exposure and the possibility of delaying biopsy. After multidisciplinary discussion, early tissue biopsy of the mediastinal lesion was prioritized.

During operation, purulent material was encountered within the mediastinal lesion, suggesting an inflammatory rather than neoplastic process. Histopathologic examination demonstrated necrotizing granulomatous inflammation with partial caseous necrosis and lymphoid follicle formation (Figure 1D). Metagenomic next-generation sequencing of the lesion identified *Bartonella henselae*, establishing the diagnosis of cat-scratch disease.

## Diagnostic assessment

The main differential diagnoses included lymphoma, tuberculosis, fungal infection, and other granulomatous inflammatory diseases. Lymphoma was initially suspected because of persistent fever, weight loss, and the necrotic mediastinal mass with airway compression. However, bone marrow examination showed no malignant infiltration, and tissue biopsy did not demonstrate neoplastic cells. Tuberculosis was also considered because of necrotizing granulomatous inflammation and mediastinal lymphadenopathy; however, there was no known tuberculosis contact or family history, and the available clinical and microbiologic findings did not support tuberculosis. Fungal infection and other granulomatous diseases were considered but were not supported by histopathology or microbiologic testing.

The combination of kitten exposure, purulent material within the mediastinal lesion, necrotizing granulomatous inflammation, detection of *Bartonella henselae* by metagenomic next-generation sequencing of tissue, and clinical-radiologic response to targeted therapy supported the diagnosis of cat-scratch disease.

The case also illustrates the limitations of relying on a single microbiologic result. Initial metagenomic testing of bronchoalveolar lavage fluid suggested *Streptococcus* species, but this finding did not explain the necrotic mediastinal lymphadenopathy or the lack of response to initial antimicrobial therapy. Tissue-based diagnosis was therefore essential.

## Therapeutic intervention

After the diagnosis of *Bartonella henselae* infection was established, the patient was treated with oral doxycycline at 2.2 mg/kg per dose twice daily for 14 days and rifampin at 10 mg/kg per dose once daily for 9 days. This regimen was chosen because of the severity of thoracic involvement and clinically significant airway compression.

Supportive care and close clinical monitoring were provided because bronchoscopy had demonstrated near-critical narrowing of the right middle lobe bronchus. No oncologic or corticosteroid therapy was administered.

## Follow-up and outcomes

Fever resolved promptly after initiation of targeted antimicrobial therapy, and cough gradually improved. During treatment, ophthalmologic examination and cranial imaging were also performed and showed no obvious abnormalities. Repeat chest computed tomography obtained after 2 weeks of treatment showed marked reduction in mediastinal lymphadenopathy, substantial relief of bronchial compression, and partial re-expansion of the affected lung (Figures 1E,F). The rapid clinical and radiologic response further supported an infectious rather than malignant process. The clinical course is summarized in Table 1.

**Table 1:** Clinical timeline of the patient.

Time point	Clinical event
Day -14 Before admission	Persistent fever, mild cough, and weight loss developed
Day -2 Early imaging	Necrotic mediastinal lymphadenopathy forming a mass-like lesion with airway compression and atelectasis in non-contrast chest CT
Day 0 Initial evaluation	Small cervical lymph nodes and decreased breath sounds over the right lung noted;
Day 4 Bronchoscopy	Severe bronchomalacia with 90% narrowing of the right middle lobe bronchus
Day 4 Microbiologic testing	BALF metagenomic testing detected Streptococcus species
Day 5 Repeat imaging	Contrast-enhanced chest CT suggested the possibility of lymphoma
Day 0–5 Initial treatment	Amoxicillin plus linezolid for 5 days; no clinical improvement
Day 5 Malignancy evaluation	Bone marrow examination unrevealing
Day 6 Mediastinal biopsy	Purulent material encountered;
Day 7 Molecular diagnosis	Metagenomic next-generation sequencing identified *Bartonella henselae*
Day 7–20 Targeted treatment	Doxycycline and rifampin administered
Day 20 Follow-up	Marked reduction in lymphadenopathy and relief of airway compression

After follow-up imaging confirmed improvement in airway compression, the child was discharged and continued to improve clinically. During 2 months of follow-up, the patient remained afebrile, respiratory symptoms did not recur, and no worsening airway obstruction was observed.

## Discussion

This case describes an unusual presentation of pediatric cat-scratch disease as necrotic mediastinal lymphadenitis mimicking malignancy and causing severe but reversible airway compression. Although cat-scratch disease typically presents with peripheral lymphadenopathy, atypical thoracic involvement can lead to diagnostic confusion, particularly when imaging demonstrates a necrotic mass-like lesion (6).

The most clinically important feature in this patient was the discrepancy between the severity of airway obstruction and the mild respiratory symptoms. Bronchoscopy showed approximately 90% luminal narrowing of the right middle lobe bronchus, yet the child did not have overt respiratory distress. This mismatch may lead to underestimation of airway risk. In children with mediastinal tumors, airway compromise is a recognized concern (8); this case shows that inflammatory mediastinal lymphadenitis from *Bartonella henselae* can produce a similar anatomic risk while remaining potentially reversible with appropriate antimicrobial therapy.

The case also highlights the diagnostic value of tissue confirmation. The initial clinical and radiologic impression strongly suggested lymphoma. However, bone marrow examination was unrevealing, and biopsy demonstrated purulent material and necrotizing granulomatous inflammation. These findings shifted the diagnostic direction from neoplastic disease toward infection. Metagenomic next-generation sequencing then identified *Bartonella henselae*, providing microbiologic confirmation (9).

Cat exposure history should be actively sought in children with unexplained necrotic lymphadenopathy, even when no inoculation papule is present. What's more, cat-scratch disease should remain in the differential diagnosis of mediastinal masses, especially when inflammatory findings coexist with imaging features that mimic malignancy. Bronchoscopy can be valuable in assessing the actual degree of airway compromise, particularly when respiratory symptoms appear mild. Finally, metagenomic sequencing may be useful when conventional testing is inconclusive, but interpretation should be integrated with the anatomic site sampled, clinical course, pathology, and treatment response (10).

Previous reports have described mediastinal or thoracic manifestations of cat-scratch disease, including necrotizing granulomatous lymphadenopathy and lesions mimicking lymphoma (5, 7). However, severe airway compression in a child appears to be rare. The rapid improvement after doxycycline and rifampin in the present case supports the reversibility of this inflammatory airway compression and underscores the importance of timely recognition (11).

Routine doxycycline use is generally avoided in children younger than 8 years. In this case, a short course was selected because of severe thoracic involvement and clinically significant airway compression. Potential dental, hepatic, and gastrointestinal adverse effects were discussed with the guardian, and safety monitoring was performed (12). Although macrolide plus rifampin or trimethoprim-sulfamethoxazole may be considered for *Bartonella henselae* infection, doxycycline plus rifampin was selected because both agents have documented activity against *Bartonella* species and are recommended or reported in severe or disseminated presentations (13, 14).

This case has several limitations. Serologic testing results for *Bartonella henselae* were not available in the current report. In addition, the follow-up period was limited, and long-term radiologic resolution could not be fully assessed. Nevertheless, the combination of exposure history, intraoperative findings, histopathology, metagenomic sequencing, and treatment response strongly supports the diagnosis.

In conclusion, *Bartonella henselae* infection may present as a necrotic mediastinal mass with critical airway compression in children. Awareness of this manifestation may help prevent delayed diagnosis, unnecessary escalation of oncologic evaluation, and underestimation of airway risk. In children with necrotic mediastinal lymphadenopathy, discordant respiratory findings, and cat exposure, cat-scratch disease should be considered, and tissue-based diagnosis should be pursued when appropriate.

## Patient perspective

Not applicable because the patient was a child. The patient's legal guardian was informed of the diagnosis, treatment course, and publication of this case report.

## Data Availability

The raw data supporting the conclusions of this article will be made available by the authors, without undue reservation.
